# How States Supported Older Adults and Persons With Disabilities During COVID-19 Through the Medicaid Program

**DOI:** 10.1093/geroni/igab046.538

**Published:** 2021-12-17

**Authors:** Edward Miller, Lisa Beauregard, Pamela Nadash, Elizabeth Simpson, Molly Wylie, Michael Gusmano

**Affiliations:** 1 University of Massachusetts Boston, Boston, Massachusetts, United States; 2 Executive Offce of Elder affairs, Executive Office of Elder Affairs, Massachusetts, United States; 3 University of Massachusetts Boston, University of Massachusetts Boston, Massachusetts, United States; 4 University of Massachusetts Boston, University of Massachusetts boston, Massachusetts, United States; 5 Rutgers University School of Public Health, Rutgers University School of Public Health, New Jersey, United States

## Abstract

COVID-19 has presented challenges for older adults who receive Medicaid home and community-based services. The federal government has allowed states to seek approval for certain flexibilities to better serve this population, including increasing provider payment rates, allowing family members to be caregivers, and permitting case management entities to provide direct services. This study uses cross-sectional data to identify factors associated with states’ adopting these Medicaid flexibilities using multivariate methods. The results indicate that the factors associated with state adoption varied depending on the flexibility. The findings suggest that states increased provider payment rates in response to prevalence of COVID-19 within their state. As cases increase, states may come under pressure to increase provider rates further which may not be feasible because of budget constraints. The results also suggest that demand for and supply of services may be a factor in whether states allowed family members to be paid caregivers. States with a higher proportion of individuals aged 85 years and older were more likely to permit caregivers to be paid which may suggest that these states may not have enough providers to care for the population. Lastly, the results suggest that provider supply was associated with whether a state allowed case management entities to provide direct care services. States with fewer home health agencies were more likely to allow this flexibility. Based on these results, states may be pursuing available Medicaid flexibilities to address provider and workforce shortages which existed prior to COVID-19 but have been exacerbated by the pandemic.

